# Risk factors associated with COVID-19 infection among Chinese elite athletes during overseas training and competitions: a large-scale cross-sectional survey

**DOI:** 10.3389/fpubh.2026.1700926

**Published:** 2026-02-09

**Authors:** Jun Chen, Jing Li, Lianmei Jin, Qirong Wang

**Affiliations:** 1 Sports Nutrition Center, National Institute of Sports Medicine, Beijing, China; 2 School of Exercise and Health, Shanghai University of Sport, Shanghai, China; 3 Health Emergency Response Center, Chinese Center for Disease Control and Prevention, Beijing, China; 4 Key Lab of Sports Nutrition, General Administration of Sport of China, Beijing, China

**Keywords:** COVID-19, cross-sectional study, elite athletes, overseas competitions, risk factors

## Abstract

**Objective:**

To analyze COVID-19 infection incidence and risk factors among Chinese national team members during overseas training and competitions (April–October 2022) to inform prevention for international events.

**Methods:**

A nationwide cross-sectional survey used electronic questionnaires distributed to 69 national teams. A total of 1,020 valid questionnaires were included for analysis, covering athletes, coaches, medical staff, team leaders, and other support personnel. The χ^2^ test was used to analyse the relationships between infection rates and variables, including individual factors, travel modes, accommodation conditions, and protective behaviors.

**Results:**

The overall infection rate was 35.5%. Infection rates differed significantly across occupational groups: athletes had the highest rate (37.9%), followed by coaches (29.8%) and team leaders (21.7%). Personnel who participated in both overseas training and competitions presented a significantly higher infection rate (47.9%) than did those who only participated in competitions (19.0%) (*p* < 0.001). Individuals vaccinated with 2 doses had the highest infection rate (48.6%), whereas those receiving ≥4 doses had the lowest infection rate (22.4%). χ^2^ analysis revealed significant risk factors for infection: sharing accommodation floors with foreign personnel, inadequate room ventilation, irregular room disinfection during training periods, taking nondirect flights, and inconsistent mask wearing.

**Conclusion:**

Infection risk among national team members abroad clustered by role, assignment type, and behaviors. Elevated risk was linked to intensive training schedules, suboptimal implementation of prevention protocols, and frequent contact with foreign personnel. Multifaceted interventions—source control, rigorous process management, and strict adherence to personal protective measures—should be strengthened to reduce infection risk.

## Introduction

The COVID-19 pandemic has exerted an unprecedented impact on the global sports system. Widespread cancelations, postponements of international events, and containment restrictions have profoundly affected athletes’ training cycles, competition schedules, and physical and mental well-being (1, 2). As the world transitions to a phase of coexistence with the virus, elite sporting competitions have gradually resumed, leading to increased cross-border training and participation by national teams (3). However, athletes of national sports delegations and their accompanying support staff (e.g., coaches, medical personnel, team leaders) represent a high-risk group for COVID-19 infection during overseas competitions and training. This elevated risk stems from factors including close contact, closed management protocols, international travel, and high physical loads (4–6). It is important to note that infection transmission within sports teams is not attributable to a single factor but rather results from the complex interplay of multi-level variables, including individual behaviors, the physical environment, and organizational policies (7).

The Social Ecological Model provides a comprehensive framework for understanding such population health issues. This model emphasizes that individual-level protective behaviors (e.g., mask-wearing, hand hygiene), environmental-level conditions of accommodation and transport (e.g., ventilation, disinfection, occupancy density), and policy-level management measures (e.g., closed-loop management, vaccination strategies) collectively form a dynamic system influencing transmission dynamics (8). Previous studies have indicated that even with regular testing and partial isolation measures, clusters of infection frequently occurred within teams in Western professional sports leagues (e.g., English Premier League, NBA), highlighting the challenges of containment in open environments (9, 10). However, most existing research has either focused on single teams or been limited to analyses of clinical characteristics, lacking a systematic assessment of the interactions between behavior, environment, and policy across the entire cross-border competition process.

During this period, Chinese national teams implemented distinctive, high-intensity prevention and control policies, characterized by strict closed-loop management, full vaccination, and health monitoring mechanisms for overseas missions under the overarching “dynamic zero-COVID” strategy (11, 12). This policy context stands in stark contrast to the more relaxed management models adopted by many Western sports leagues, thereby offering a unique setting to identify truly effective protective and risk factors within a highly compliant population operating under stringent measures. Furthermore, while the multidimensional impact of prolonged pandemic control on athlete behavior and health—such as physical activity, nutrition, sleep, and psychological state—has garnered scholarly attention (13, 14), there remains a scarcity of empirical evidence, supported by nationally representative data, on how to systematically assess and optimize intervenable risk factors during high-risk cross-border missions.

To address this gap, a national multi-center cross-sectional survey was launched in November 2022 through a collaboration between the General Administration of Sport of China and the Chinese Center for Disease Control and Prevention. This study aims to analyze the incidence of SARS-CoV-2 infection and its key influencing factors among national team members during overseas training and competition between April and October 2022. By focusing on various dimensions, including personnel categories, travel modes, accommodation conditions, and protective behaviors, this research seeks to identify high-risk links and modifiable factors. The findings are intended to provide data-driven evidence and a theoretical basis for formulating scientific and precise prevention and control strategies for Chinese sports delegations participating in future international major events, such as the Asian Games and Olympic Games.

## Methods

### Study design

This study employed a nationwide, cross-sectional survey design in which a structured questionnaire was used to collect systematic data on COVID-19 infection status and associated behavioral and environmental risk factors among Chinese national sports team members during periods of overseas training and competition. The questionnaire addressed multiple dimensions, including travel arrangements, personal protective behaviors, accommodation and catering conditions, vaccination status, and infection history. The survey was jointly initiated by the General Administration of Sport of China and the Chinese Center for Disease Control and Prevention in November 2022. Its primary objective was to assess the risk of COVID-19 infection faced by national team members during overseas assignments conducted between April and October 2022. The study protocol was approved by the Ethics Committee of the Institute of Sports Medicine, General Administration of Sport of China (Approval No.: 202207). Electronic informed consent was obtained from all participants before they proceeded with the survey.

### Study subjects and inclusion criteria

The study population consisted of individuals affiliated with national sports teams who participated in overseas training, overseas competitions, or both during the period from April 1 to October 31, 2022. The participants were classified into five categories: (1) registered national team athletes, (2) national team coaches, (3) accompanying medical personnel, (4) team leaders, and (5) other support staff (e.g., logistics personnel, interpreters, and security officers).

Inclusion criteria:

1. Official affiliation with a Chinese national-level team and participation in at least one overseas training or competition assignment during the designated study period.
2. Direct involvement in training, competition, or relevant support tasks while abroad.
3. Provision of informed consent and successful completion of the questionnaire.

Exclusion criteria:

1. Reserve or alternate personnel who did not undertake overseas assignments.
2. Questionnaires lacking critical variables (e.g., infection status or assignment details).
3. Suspected duplicate submissions or responses with unverifiable identity information.

### Data collection instrument and content

The “Survey on Risk Factors for SARS-CoV-2 Infection in National Teams during Overseas Training and Competition” was designed in line with established COVID-19 prevention protocols and epidemiological principles. Following its development, the draft questionnaire was reviewed by an expert panel in sports medicine, epidemiology, and public health. It was then piloted with a national team to evaluate item clarity, comprehensibility, and time requirement. Final adjustments to the wording were made based on the pilot feedback. The questionnaire comprises six major sections: (1) Basic information: gender, age, affiliated team, occupational role, and vaccination status. (2) Overseas assignment arrangements: modes of departure and return transportation, transit details, and accommodation arrangements. (3) Training and competition behaviors: Contact with foreign personnel, dining habits, and personal protective behaviors (e.g., mask use, room ventilation, room disinfection). (4) Operational definition of infection: A case was operationally defined as an individual with a self-reported COVID-19 diagnosis during the overseas mission, corroborated by either the team physician or a local healthcare provider and confirmed through a positive result on a nucleic acid amplification test (e.g., RT-PCR) or a rapid antigen test. (5) Close Contact Management: Identification as close contact, isolation measures undertaken, and subsequent infection status. (6) Implementation of prevention measures: participation in organized prevention and control activities and adherence to standardized protective protocols.

### Data collection and quality control

Electronic questionnaires were distributed to national team members through team management personnel, who facilitated questionnaire completion and submission. A total of 1,056 questionnaires were distributed, and 1,020 valid responses were received, yielding a response rate of 96.6%. The valid sample included 642 athletes, 181 coaches, 73 medical personnel, 46 team leaders, and 78 other support staff. All the responses were uniformly coded and entered by trained members of the research team. To ensure data integrity, 10% of the entries were randomly selected for cross-verification of consistency and accuracy.

### Statistical analysis

All statistical analyses were performed using SPSS 26.0 (IBM Corp., Armonk, NY, United States). Categorical variables, such as gender and occupational category, were described using frequencies (n) and percentages (%). Between-group comparisons were conducted using the Chi-square test. To quantify the strength of association between potential risk factors and infection outcomes, odds ratios (ORs) with 95% confidence intervals (CIs) were calculated. All statistical tests were two-sided, and a *p*-value < 0.05 was considered statistically significant.

## Results

### Overall infection status

Among the 1,020 national team members who participated in overseas assignments between April and October 2022, a total of 362 individuals reported SARS-CoV-2 infections, yielding an overall infection rate of 35.5%. Among these cases, 12 were reinfections, representing a reinfection rate of 3.31% (12/362). The infection rates varied substantially across different sports teams (Supplementary Table S1). For instance, extremely high infection rates were observed in the Youth Men’s Volleyball team (100%, 20/20), the Men’s 3×3 Basketball team (94.4%, 17/18), and the U17 Women’s Football team (89.2%, 33/37). In contrast, significantly lower rates were recorded in the Shooting team (2.7%, 2/74) and the Diving team (0.0%, 0/20).

### Association of demographic, mission, and vaccination characteristics with infection rate

As shown in Table 1, a significant difference in infection rate was observed across age groups (χ^2^ = 18.365, *p* = 0.005). The adolescent group aged 10–19 years exhibited the highest infection rate (47.3%), while the group aged 40–49 years had the lowest (25.5%). Meanwhile, no statistically significant difference in infection rate was found between genders (χ^2^ = 3.261, *p* = 0.071). Regarding occupational category, athletes (37.9%) and other staff members (39.7%) showed relatively higher infection rates, whereas team leaders had the lowest rate (21.7%); however, the difference among these groups did not reach statistical significance (χ^2^ = 8.725, *p* = 0.068).

**Table 1:** Basic characteristics and infection rates of national team members during overseas training and competition assignments.

Variable		Cases/total	Infection rate (%)	χ^2^ value	*P* value
Age (years)	0–9	1/3	33.3	18.365	0.005
	10–19	96/203	47.3		
	20–9	164/477	34.4		
	30–39	52/163	31.9		
	40–49	25/98	25.5		
	50–59	20/62	32.3		
	≥60	4/14	28.6		
Gender	Male	199/599	33.2	3.261	0.071
	Female	163/421	38.7		
Occupational category	Athletes	243/642	37.9	8.725	0.068
	Coaches	54/181	29.8		
	Team leaders	10/46	21.7		
	Medical staff	24/73	32.9		
	Other support staff	31/78	39.7		
Assignment type	Competition	83/437	19.0	90.885	< 0.001
	Training + competition	279/583	47.9		
Vaccination doses	Unvaccinated	22/70	31.4	25.903	< 0.001
	1 doses	2/10	20.0		
	2 doses	84/173	48.6		
	3 doses	220/615	48.6		
	≥4 doses	34/152	22.4		

The type of mission assignment was a strong predictor of infection risk. Personnel engaged in combined training and competition assignments had a significantly higher infection rate compared to those involved solely in competition assignments (47.9% vs. 19.0%; OR = 3.91, 95% CI: 2.93–5.23, *p* < 0.001).

Notably, a significant difference in infection rate was observed based on the number of vaccine doses received (χ^2^ = 25.903, *p* < 0.001). Individuals who had received two doses had the highest infection rate (48.6%), while those who had received four or more doses exhibited the lowest infection rate (22.4%).

### Analysis of risk factors for infection in different personnel categories athletes and coaches

As shown in Table 2, the chosen mode of travel significantly influenced the risk of infection among athletes and coaches. Those who took non-direct flights had a significantly higher infection rate than those on direct flights (46.8% vs. 28.9%; OR = 2.18, 95% CI: 1.64–2.90, *p* < 0.001). Upon arrival, individuals using chartered vehicles to reach the training/competition venue had a higher infection rate (41.2%) compared to those using airplanes (34.1%) or trains (30.8%). For the return journey, the infection rate was higher among those using commercial flights compared to those returning via charter flights (38.1% vs. 26.4%; OR = 1.73, 95% CI: 1.14–2.63, *p* = 0.009). Dining (46.1% vs. 28.8%; OR = 2.12, 95% CI: 1.61–2.79, *p* < 0.001) or shopping (49.0% vs. 30.1%; OR = 2.24, 95% CI: 1.69–2.98, *p* < 0.001) during layovers were both associated with a significantly increased risk of infection.

**Table 2:** Infection rates among athletes and coaches by travel-related variables.

Variable				Infection status			Positivity rate (%)	χ^2^ value	*P* value
				Positive	Negative	Total			
Outbound travel	Transportation to destination	Direct flight	Yes	142	350	492	28.9	27.692	0.000
			No	155	176	331	46.8		
		Airplane	Not selected	168	230	398	42.2	4.900	0.027
			Selected	107	207	314	34.1		
		Chartered coach	Not selected	41	103	144	28.5	7.847	0.005
			Selected	234	334	568	41.2		
	Overnight accommodation during transit		Yes	108	197	305	35.4	2.325	0.127
			No	167	240	407	41.0		
Return travel	Return flight type		Chartered flight	37	103	140	26.4	6.824	0.009
			Commercial flight	260	423	683	38.1		
	Dining during layover		Yes	160	187	347	46.1	26.127	0.000
			No	137	339	476	28.8		
	Shopping during layover		Yes	128	133	261	30.1	27.811	0.000
			No	169	393	562	36.4		
Competition	Dining with foreign personnel		Yes	236	304	540	43.7	39.459	0.000
			No	61	222	283	21.6		
	Accommodation building		Separate building	22	107	129	17.1	24.028	0.000
			Shared with foreign personnel	275	419	694	39.6		
	Daily room ventilation		>30 min	252	496	748	33.7	20.458	0.000
			<30 min	45	30	75	60.0		

Regarding behaviors and environment during the competition period, participants in team sports had a substantially higher infection rate than those in individual sports (57.5% vs. 20.6%; OR = 5.19, 95% CI: 3.71–7.26, *p* < 0.001). Dining with foreign personnel was associated with a significantly higher infection rate compared to having no such history (43.7% vs. 21.6%; OR = 2.87, 95% CI: 1.87–4.41, *p* < 0.001). Individuals sharing accommodation buildings with foreign personnel had a higher infection rate than those housed in separate buildings (39.6% vs. 17.1%; OR = 3.21, 95% CI: 1.96–5.25, *p* < 0.001). Inadequate room ventilation, defined as less than 30 min daily, was associated with a significantly higher infection rate compared to sufficient ventilation (60.0% vs. 33.7%; OR = 2.95, 95% CI: 1.79–4.87, *p* < 0.001). Notably, infection rates among those who never wore masks in areas such as locker rooms (83.3%), waiting areas (82.8%), and check-in areas (52.9%) were significantly higher than those who consistently wore masks in these respective settings.

### Medical staff

As shown in Table 3, infection risk among medical staff was closely associated with the nature of their work and environmental exposure. Those involved in the team’s COVID-19 prevention work but with inadequate personal protective equipment had a significantly higher infection rate than those with standardized protection (44.44% vs. 10.00%; OR = 7.20, 95% CI: 0.84–61.83, *p* = 0.027).

**Table 3:** Infection rates among medical staff by accommodation and exposure variables.

Variable			Infection status			Positivity rate (%)	χ^2^ value	*P* value
			Positive	Negative	Total			
During competition	Accommodation type	Single room	2	30	32	6.25	7.347	0.025
		Double room	8	30	38	21.05		
		Other room type	2	1	3	66.67		
	Building arrangement	Separate building	0	8	8	0	0.679	0.41
		Building shared with foreign personnel	12	56	68	17.65		
	Coresidence on same floor as foreign personnel	Yes	12	45	57	21.05	6.572	0.037
		No	0	8	8	0		
During training	Accommodation type	Single room	10	9	19	52.63	12.045	0.002
		Double room	1	18	19	5.26		
		Other room type	0	3	3	0		
	Building arrangement	Separate building	2	7	9	22.22	0	1
		Building shared with foreign personnel	9	23	32	28.13		
	Room disinfection frequency	Regular	4	21	25	16.0	9.027	0.011
		Irregular	7	5	12	58.33		
		None	0	4	4	0		
	Contact with foreign personnel	Yes	6	9	15	40.0	5.977	0.05
		No	3	20	23	13.04		

During international competitions, the infection rate was higher among those residing in double-occupancy rooms compared to single-occupancy rooms (21.05% vs. 6.25%; OR = 3.94, 95% CI: 0.79–19.59, *p* = 0.025). Individuals sharing the same floor with foreign personnel had a significantly higher infection rate (21.05%) compared to those residing on different floors (0%; none of whom were infected).

During international training periods, the risk pattern shifted. Notably, the infection rate was substantially higher among those in single-occupancy rooms compared to double-occupancy rooms (52.63% vs. 5.26%; OR = 20.00, 95% CI: 2.38–168.17, *p* = 0.002). Irregular room disinfection was associated with a significantly higher infection rate compared to regular disinfection (58.33% vs. 16.00%; OR = 7.29, 95% CI: 1.72–30.87, *p* = 0.011). Furthermore, contact with foreign personnel during training was associated with a higher infection rate compared to no such contact (40.00% vs. 13.04%; OR = 4.44, 95% CI: 1.01–19.58, *p* = 0.050).

### Team leaders and other support staff

As shown in Table 4, infection risk in this population was highly associated with travel and accommodation arrangements. The infection rate was significantly higher among those who took non-direct flights on their outbound journey compared to those on direct flights (50.0% vs. 23.1%; OR = 3.33, 95% CI: 1.60–6.94, *p* = 0.002). A consistent trend was observed for the return journey (46.3% vs. 26.5%; OR = 2.43, 95% CI: 1.17–5.05, *p* = 0.027).

**Table 4:** Infection rates among team leaders and support staff by exposure variables.

Variable			Infection status			Positivity rate (%)	χ^2^ value	*P* value
			Positive	Negative	Total			
Outbound travel	Direct flight	Yes	60	18	78	23.1	9.477	0.002
		No	23	23	46	50.0		
	Used other transport (airport → venue)	Yes	76	40	116	34.5	1.634	0.201
		No	7	1	8	12.5		
	Overnight transit accommodation	Yes	23	10	33	30.3	0.357	0.550
		No	53	30	83	36.1		
Return travel	Direct flight	Yes	61	22	83	26.5	4.879	0.027
		No	22	19	41	46.3		
	Used other transport (venue → airport)	Yes	72	39	111	35.1	2.051	0.152
		No	11	2	13	15.4		
	Overnight transit accommodation	Yes	28	13	41	31.7	27.811	0.000
		No	44	26	70	37.1		
	Dined during layover	Yes	37	24	61	39.3	2.139	0.114
		No	46	17	63	27.0		
	Shopped during layover	Yes	22	11	33	33.3	0.001	0.969
		No	61	30	91	33.0		
During competition	Room type	Single	42	22	64	34.4	0.103	0.749
		Double	41	19	60	31.7		
	Building arrangement	Dedicated building	16	1	17	5.9	6.577	0.010
		Shared with internationals	67	40	107	37.4		
	Same-floor coresidence (Int’l)	Yes	61	35	96	36.5	0.341	0.559
		No	6	5	11	45.5		
	Daily room ventilation	≥30 min	80	35	115	30.4	4.951	0.026
		<30 min	3	6	9	66.7		
	Room cleaning frequency	Regular	33	23	56	41.1	3.177	0.204
		As-needed	30	12	42	28.6		
		None	20	6	26	23.1		
	Shared vehicle with Int’l	Yes	26	22	48	45.8	5.144	0.023
		No	52	18	70	25.7		
	Mask use at events	Always	41	22	63	34.9	3.525	0.172
		Often	0	1	1	100.0		
		Occasionally	0	1	1	100.0		
	Group photos with Int’l	Yes	33	12	45	26.7	1.306	0.253
		No	50	29	79	36.7		
	Close interaction at ceremonies	Yes	18	11	29	37.9	2.723	0.099
		No	9	1	10	10.0		
During training	Room type	Single	20	17	37	45.9	0.159	0.690
		Double	24	17	41	41.5		
	Building arrangement	Dedicated building	14	3	17	17.6	5.950	0.015
		Shared with Internationals	30	31	61	50.8		
	Daily room ventilation	≥30 min	44	32	76	42.1	2.656	0.103
		<30 min	0	2	2	100.0		
	Room cleaning frequency	Regular	18	20	38	52.6	9.106	0.011
		As-needed	16	14	30	46.7		
		None	10	0	10	0.0		
	Shared vehicle with Int’l	Yes	9	18	27	66.7	8.797	0.003
		No	33	15	48	31.3		
	Mask use to venue	Yes	40	32	72	44.4	0.144	0.704
		No	2	1	3	33.3		

Int’l, International personnel.

During international competitions, individuals sharing the same accommodation building with foreign personnel had a significantly higher infection rate than those housed in separate buildings (37.4% vs. 5.9%; OR = 9.55, 95% CI: 1.21–75.28, *p* = 0.010). An average daily room ventilation duration of less than 30 min was associated with a higher infection rate compared to adequate ventilation (66.7% vs. 30.4%; OR = 4.57, 95% CI: 1.05–19.87, *p* = 0.026). Sharing transportation with foreign personnel to the competition venues was associated with a higher infection rate compared to not sharing vehicles (45.8% vs. 25.7%; OR = 2.44, 95% CI: 1.16–5.13, *p* = 0.023).

During international training periods, sharing the same accommodation building with foreign personnel (50.8% vs. 17.6%; OR = 4.83, 95% CI: 1.25–18.66, *p* = 0.015) and sharing transportation with them (66.7% vs. 31.3%; OR = 4.44, 95% CI: 1.67–11.82, *p* = 0.003) remained significant risk factors. Furthermore, the infection rate was higher among individuals whose rooms were cleaned regularly by hotel staff (52.6%) compared to those whose rooms were not serviced (0%; no infections).

### Exposure to high-risk behaviors during training and competition

A comprehensive analysis of behavioral and environmental exposures among all personnel during training and competition periods revealed multiple factors significantly associated with infection risk. Participation in team sports events constituted a strong risk factor, with a significantly higher infection rate compared to individual events (57.5% vs. 20.6%; OR = 5.19, 95% CI: 3.71–7.26, *p* < 0.001). Protective behaviors in functional competition areas were crucial. The infection rate among those who never wore masks in locker rooms was significantly higher than among those who always wore masks (83.3% vs. 34.3%; OR = 9.33, 95% CI: 2.55–34.11, *p* < 0.001). Similar significant trends were observed in waiting areas (OR = 9.78, 95% CI: 3.86–24.77, *p* < 0.001) and check-in areas (OR = 2.71, 95% CI: 1.05–6.99, *p* = 0.040). Poor environmental ventilation was another key risk; individuals with an average daily room ventilation duration of less than 30 min had a significantly higher infection rate than those with adequate ventilation (60.0% vs. 33.7%; OR = 2.95, 95% CI: 1.79–4.87, *p* < 0.001).

Frequent and close-contact behaviors significantly increased infection risk, including dining with foreign personnel (OR = 2.87, 95% CI: 1.87–4.41, *p* < 0.001), sharing vehicles with them (OR = 2.44, 95% CI: 1.16–5.13, *p* = 0.023), sharing the same accommodation floor/building with them (OR = 3.21, 95% CI: 1.96–5.25, *p* < 0.001), and taking group photos (OR = 1.47, 95% CI: 1.11–1.96, *p* = 0.008). Furthermore, during training periods, the infection risk was 3.6 times higher for those with irregular room disinfection compared to regular disinfection (OR = 3.64, 95% CI: 1.67–7.94, *p* = 0.001). During competitions, behaviors such as wiping sweat and rubbing eyes were also associated with a significantly elevated infection rate (OR = 4.92, 95% CI: 3.41–7.10, *p* < 0.001).

## Discussion

This study provides a systematic analysis of SARS-CoV-2 infections among members of Chinese national sports teams during overseas training and competition assignments in 2022 on the basis of a nationwide survey jointly conducted by the General Administration of Sport of China and the Chinese Center for Disease Control and Prevention. The findings indicate a substantial overall infection rate of 35.5% among personnel participating in international assignments, influenced by multiple factors, including assignment type, accommodation conditions, protective behaviors, contact patterns, and vaccination status. Combined training and competition assignments, residences in double rooms, shared accommodation floors with foreign personnel, irregular room disinfection, direct contact with foreign individuals, and inconsistent adherence to personal protective measures were identified as independent risk factors. In contrast, receiving four or more doses of the COVID-19 vaccine had a significant protective effect.

This study observed an association between receiving ≥4 vaccine doses and a lower risk of infection. This inverse correlation aligns with global evidence supporting the protective effect of booster doses (15, 16). However, as an observational study, this association must be interpreted with caution. Residual confounding may exist due to differences in risk behaviors, exposure opportunities, and timing of vaccination among individuals with different vaccination doses. For instance, the highest infection rate among those who received two doses might reflect the combined effect of waning natural immunity over time and subsequent exposure during high-risk assignments. Therefore, while we consider vaccination an important protective correlate, its precise protective efficacy warrants further confirmation through prospective studies.

The finding of a higher infection risk among individuals residing in single rooms during training periods is particularly noteworthy and warrants discussion. One plausible explanation lies in behavioral pattern differences: individuals in single rooms might perceive their private space as safe, potentially leading to relaxed vigilance in common areas (e.g., corridors, gyms, dining halls) and lapses in protective measures. In contrast, roommates in double rooms might form a mutually reinforcing “protective bubble,” reducing unnecessary external contact. Alternatively, room assignment itself could be a confounder: individuals in critical roles (e.g., team doctors, key support staff) might be allocated single rooms, but their job responsibilities inherently require frequent movement across different zones and contact with more people, thereby increasing their exposure risk. Furthermore, the possibility that some single rooms had poorer physical ventilation than double rooms, increasing the risk of aerosol accumulation, cannot be ruled out (17, 18). These speculations collectively suggest that room type alone is not the determinant of risk; rather, the coupled behavioral patterns, personnel mobility, and environmental ventilation characteristics jointly shape infection outcomes. The uncertainty surrounding this counterintuitive finding highlights the need for future research incorporating detailed behavioral observations and environmental monitoring to clarify the underlying mechanisms.

Multivariable analysis identified non-direct flights, non-standard mask-wearing, and sharing accommodation floors with foreign personnel as independent risk factors for infection. These findings share commonalities with reports from international professional sports domains. For example, studies of the South African rugby league (19), European football leagues (20), and the NBA (21) similarly identified intensive travel schedules and shared living environments as key drivers of team clusters. However, our study was conducted against the backdrop of China’s strict “closed-loop management” policy, indicating that even within a highly controlled system, the virus could still infiltrate through essential cross-border travel and unavoidable contact points. The substantially elevated risk associated with combined “training and competition” assignments underscores the cumulative exposure effect of prolonged overseas training, contrasting sharply with the risk profile of short-term competition participation.

The standardization of protective behaviors directly determined infection outcomes (22). This study found that non-standard mask-wearing and irregular disinfection significantly increased infection risk. Beyond operational issues, psychological factors crucially influence behavioral adherence (23). Elite athletes and support staff undergoing long-term, enclosed, and high-pressure overseas assignments are highly susceptible to “behavioral fatigue” and “psychological burnout,” leading to diminished motivation for protection (24). Younger athletes, in particular, might underestimate their personal infection risk due to “optimism bias” (25). Research by Taheri et al. notes the complex impact of prolonged pandemic control measures on athletes’ psychological states and health behaviors (13, 14); such changes may indirectly weaken their willingness and quality in implementing protective measures. Consequently, future prevention strategies must move beyond simply issuing directives. They should integrate insights from behavioral science and psychology, employing effective communication, supportive environments, and regular psychological adjustment to sustain long-term protective adherence within teams.

This study also has limitations. First, reliance on self-report questionnaires introduces the potential for recall bias and reporting bias, particularly concerning contact behaviors and self-assessment of protection standardization. Second, the absence of biological validation (e.g., viral sequencing, antibody testing) hinders definitive identification of viral sources and transmission chains. Third, small sample sizes in some subteams (<10 individuals) may introduce selection bias for specific infection rate estimates, warranting cautious interpretation. Additionally, the survey was conducted in November 2022 and may not reflect infection risks evolving under the subsequent dominance of variants such as the XBB sublineages in 2023. Future research should incorporate longitudinal tracking for dynamic risk assessment and intervention evaluation.

## Conclusion

Leveraging large-scale, nationwide data from Chinese national team members, this study provides the first systematic identification of key risk factors for SARS-CoV-2 infection throughout the entire process of overseas competitions and training assignments. These findings enrich the evidence base for public health prevention and control strategies targeting highly mobile populations. The findings underscore the necessity of developing a multilayered, process-oriented, and actionable prevention strategy framework centered on “mission workflow - behavioral standardization - environmental management.” This approach is critical for enhancing the health resilience of elite sport systems and their capacity to respond effectively during international sporting events.

## Data Availability

The original contributions presented in the study are included in the article/supplementary material, further inquiries can be directed to the corresponding author.
