# Supplementary material for: Risk factors associated with COVID-19 infection among Chinese elite athletes during overseas training and competitions: a large-scale cross-sectional survey
Source: Front Public Health. 2026 Feb 9;14:1700926. doi: 10.3389/fpubh.2026.1700926 (PMC12926398; doi:10.3389/fpubh.2026.1700926)
Supplement: Supplementary file 1 [file Table_1.docx]

**S1. Statistical Summary of Infections Among National Team Delegations Participating in Overseas Training and Competitions**

| No. | Delegation / Team composition | Total participants | Number of infections | Infection rate (%) |
| --- | --- | --- | --- | --- |
| 1 | Women's Field Hockey Team | 1 | 1 | 100.0 |
| 2 | Modern Pentathlon Team | 1 | 1 | 100.0 |
| 3 | Men's Sprint Group | 3 | 3 | 100.0 |
| 4 | Men's Field Hockey Team | 1 | 1 | 100.0 |
| 5 | Junior Men's Volleyball Team | 20 | 20 | 100.0 |
| 6 | Men's 3x3 Basketball Team | 18 | 17 | 94.4 |
| 7 | Additional Team - U17 Women's Football Team | 37 | 33 | 89.2 |
| 8 | Junior Women's Volleyball Team | 16 | 14 | 87.5 |
| 9 | Women's 3x3 Basketball Team | 19 | 16 | 84.2 |
| 10 | Men's Volleyball Team | 26 | 21 | 80.8 |
| 11 | National Track and Field Team (Randy Group) | 4 | 3 | 75.0 |
| 12 | Women's Volleyball Team | 19 | 14 | 73.7 |
| 13 | Sport Climbing Team | 22 | 16 | 72.7 |
| 14 | Judo Team | 35 | 24 | 68.6 |
| 15 | 3x3 Basketball U21 Team | 24 | 16 | 66.7 |
| 16 | National Rowing Team (Tall Athletes Group) | 6 | 4 | 66.7 |
| 17 | Additional Team - Women's Basketball U18 Team | 21 | 14 | 66.7 |
| 18 | Shooting (Shotgun) Team | 27 | 16 | 59.3 |
| 19 | Track and Field Jumping Team (Yang Zhongmin Group) | 9 | 5 | 55.6 |
| 20 | Track and Field Hurdles Team (Hu Hao Group) | 13 | 7 | 53.8 |
| 21 | Men's Sabre Fencing Team | 2 | 1 | 50.0 |
| 22 | Additional Team - Triathlon Team | 2 | 1 | 50.0 |
| 23 | Boxing Team | 7 | 3 | 42.9 |
| 24 | Swimming Team 2 | 12 | 5 | 41.7 |
| 25 | Women's Basketball Team | 12 | 5 | 41.7 |
| 26 | Rowing Team | 59 | 24 | 40.7 |
| 27 | Chinese Athletics Delegation | 10 | 4 | 40.0 |
| 28 | Additional Team - U21 Men's Football Team | 10 | 4 | 40.0 |
| 29 | Youth Men's Volleyball Team | 18 | 7 | 38.9 |
| 30 | Swimming Team 1 | 26 | 10 | 38.5 |
| 31 | Track Cycling Team | 33 | 11 | 33.3 |
| 32 | Men's Basketball Team | 3 | 1 | 33.3 |
| 33 | Table Tennis Team (First & Second Squads) | 10 | 3 | 30.0 |
| 34 | Rhythmic Gymnastics Team | 4 | 1 | 25.0 |
| 35 | Weightlifting Team | 13 | 3 | 23.1 |
| 36 | Women's Volleyball Team | 9 | 2 | 22.2 |
| 37 | National Track and Field Team (Women's Sprint & Hurdles) | 5 | 1 | 20.0 |
| 38 | Track and Field Jumping Team (Pole Vault) | 5 | 1 | 20.0 |
| 39 | Taekwondo Team | 31 | 6 | 19.4 |
| 40 | Wrestling Team | 59 | 11 | 18.6 |
| 41 | Additional Team - Breaking Team | 11 | 2 | 18.2 |
| 42 | Table Tennis Team | 6 | 1 | 16.7 |
| 43 | Table Tennis Team | 23 | 3 | 13.0 |
| 44 | Marathon Team | 18 | 2 | 11.1 |
| 45 | Sport Climbing Team | 18 | 1 | 5.6 |
| 46 | Track Cycling Team | 31 | 1 | 3.2 |
| 47 | Additional Team - National Shooting Team | 74 | 2 | 2.7 |
| 48 | National Youth Beach Volleyball Team | 8 | 0 | 0.0 |
| 49 | Canoe Slalom Team | 3 | 0 | 0.0 |
| 50 | Canoe Sprint Team | 24 | 0 | 0.0 |
| 51 | Surfing Team | 1 | 0 | 0.0 |
| 52 | Track and Field Throwing Team (Hammer Throw Foreign Coach Group) | 6 | 0 | 0.0 |
| 53 | Rhythmic Gymnastics Team | 8 | 0 | 0.0 |
| 54 | Track and Field Middle & Long Distance Team (Shi Peng Group) | 3 | 0 | 0.0 |
| 55 | Men's Foil Fencing Team and Support Staff | 5 | 0 | 0.0 |
| 56 | Table Tennis First Team | 16 | 0 | 0.0 |
| 57 | Men's Decathlon Team | 5 | 0 | 0.0 |
| 58 | Track and Field Hurdles Group (Wen Jun Group) | 13 | 0 | 0.0 |
| 59 | Men's Basketball U18 Team | 18 | 0 | 0.0 |
| 60 | Figure Skating Team | 9 | 0 | 0.0 |
| 61 | Table Tennis Second Team | 4 | 0 | 0.0 |
| 62 | Table Tennis National Team (First & Second Squads) | 3 | 0 | 0.0 |
| 63 | Youth Beach Volleyball Team | 8 | 0 | 0.0 |
| 64 | Road Cycling Team | 3 | 0 | 0.0 |
| 65 | Figure Skating Team | 10 | 0 | 0.0 |
| 66 | Diving Team | 20 | 0 | 0.0 |
| 67 | Mountain Bike Team | 8 | 0 | 0.0 |
| 68 | Chinese Chess Team | 7 | 0 | 0.0 |
| 69 | Additional Team - International Wushu Federation (Zhang Qiuping Team) | 5 | 0 | 0.0 |
|  | **Overall Total** | **1020** | **362** |  |
